# Effects of an Aqueous Extract of Dangguijagyagsan on Serum Lipid Levels and Blood Flow Improvement in Ovariectomized Rats

**DOI:** 10.1155/2014/497836

**Published:** 2014-09-07

**Authors:** In Sil Park, Hye Won Lee, Jin Ah Ryuk, Byoung Seob Ko

**Affiliations:** Korean Medicine Based Herbal Drug Development Group, Herbal Medicine Research Division, Korea Institute of Oriental Medicine, 1672, Yuseong-daero, Daejeon 305-811, Republic of Korea

## Abstract

Dangguijagyagsan (DJS), a traditional herbal prescription, has long been used to treat menopause-related symptoms. We identified the cardioprotective effects of an aqueous extract of DJS using an ovariectomized (OVX) and ferric chloride- (FeCl-) induced carotid thrombosis rat model. Female Sprague-Dawley (SD) rats were ovariectomized or Sham-operated (Sham-control). The ovariectomized rats were divided into three groups: OVX with saline (OVX-control), aspirin 30 mg/kg/day (OVX-ASA), and DJS 100 mg/kg/day (OVX-DJS). The treatments were administered for 5 weeks. Then, blood samples were collected to analyze the serum lipid levels and platelet aggregation. The topical application of 40% FeCl_3_ induced intravascular thrombosis, which was used to test thrombotic occlusion and for histological examination. Body weight and the levels of total cholesterol (TC), triglyceride (TG), and LDL-cholesterol (LDL-C) increased in the OVX rats. These effects were reduced by ASA and DJS treatment. In addition, ASA and DJS treatment significantly inhibited platelet aggregation. These treatments also increased time to occlusion and decreased both thrombus size and the presence of collagen fibers in surrounding vessel walls compared with the Sham-control and OVX-control groups. These results suggest that DJS has beneficial effects in terms of preventing cardiovascular disease in menopausal woman because it can reduce the serum lipid levels and improve blood flow by inhibiting platelet aggregation and thrombus formation.

## 1. Introduction

The incidence of cardiovascular disease (CVD) remains the leading cause of death, with high morbidity and mortality after menopause. Estrogen deficiency may cause an increase in cardiovascular risks by affecting lipoprotein metabolism, platelet aggregation ability, and vessel resistance. It is well established that the incidence of CVD is due to hyperlipidemia, a condition characterized by significant increases in total cholesterol (TC), triglycerides (TG), and low-density lipoprotein cholesterol (LDL-C) and a reduction in high-density cholesterol (HDL-C) [1, 2]. Platelets from hypercholesterolemic patients were more reactive to aggregating reagents such as epinephrine and adenosine diphosphate (ADP) than were platelets from normal individuals, and platelets from hypercholesterolemic patients showed hyperactivity and shortened platelet survival* ex vivo* [3]. Estrogen deficiency in OVX animals and menopausal woman can cause activated platelet aggregation and thrombosis by affecting platelet agonist hydrolysis and nitric oxide production in blood vessels. An atherosclerotic plaque rupture releases collagen, lipids, and smooth muscle cells and leads to platelet activation and occlusive thrombosis, thereby stimulating the coagulation cascade system. Thus, abnormal platelet activation plays a key role in interrupting blood flow, for which antiplatelet drugs are available to prevent intravascular thrombosis [4]. Hormone replacement therapy (HRT) is the combination treatment with estrogen or progestin. As a primary therapy, HRT has been observed to exert a protective effect against menopausal cardiovascular disease by modulating the serum lipid profiles and augmenting the response to atherosclerosis [5]. However, HRT studies in menopausal women have reported side effects such as breast cancer, thromboembolic disease, and ischemic stroke [6]. Considering the negative side effects, herbal medicines have been given further attention as candidates for alternative therapy for safe and effective HRT [7]. Dangguijagyagsan (DangQui-Shaoyao-San in Chinese; Tokishakuyakusan in Japanese) is a traditional medicinal prescription that has long been used for the treatment of menopause-related symptoms in East Asia. Previous gynecologic reports related to DJS have documented its improvement of ovarian hormones [8], estrogenic action [9], and neurotoxicity effects of DJS under postmenopausal conditions [10]. However, no studies have investigated the effectiveness of DJS in restoring blood flow. In this study, aspirin was used as a positive control. Aspirin is a widely used anti-thrombosis drug, and low-dose aspirin has been shown to have a preventive effect on thrombus formation in animal testing [11]. Thus, we aimed to investigate the oral administration of DJS (100 mg/kg/day) for 5 weeks to determine the beneficial effects on serum lipid levels and blood flow improvement in OVX and thrombosis-induced rat models in comparison with a Sham-control group.

## 2. Materials and Methods

### 2.1. Chemicals and Reagents

Acetonitrile (100%), formic acid (99.9%), and distilled water were purchased from J.T. Baker (HPLC grade, USA). Reference compounds of albiflorin and paeoniflorin were purchased from Wako (Japan). *z*-Ligustilide was purchased from ChromaDex (USA). Nodakenin and decursin were divided from Ministry of Food and Drug Safety (Korea).

### 2.2. Plant Extracts

The formula of DJS consists of 6 herbs, including* Paeonia lactiflora* (93.75 g),* Cnidium officinale* (56.25 g),* Alisma orientale* (56.25 g),* Angelica gigas* (28.13 g),* Poria cocos* (28.13 g), and* Atractylodis rhizoma alba* (28.13 g). Briefly, 290 g of the 6-herb mixture was boiled in 6 L of distilled water for 4 h at 100°C and filtered using filter paper (Whatman, Maidstone, UK). After filtration, the extract was evaporated and lyophilized. The DJS product (KIOM PH 130005) was stored at Korea Institute of Oriental Medicine (KIOM, Daejeon, Korea) until used in this experiment. The composition of DJS extract and contents of ingredients are shown in Table 1.

### 2.3. Chromatographic Conditions of HPLC-DAD

The contents albiflorin, paeoniflorin,* z*-ligustilide, decursin, and nodakenin in the DJS water extract were analyzed using an HPLC instrument (Agilent Technologies, USA) with a Atlantis dC_18_ column (4.6 × 250 mm, 5 *μ*m; Waters, USA). The mobile phase consisted of the solvents, distilled water (A) and acetonitrile with 0.1% formic acid (B). The following gradient was used: 0 min, A : B 90 : 10 (v/v); 20 min, A : B 75 : 25; 25 min, A : B 75 : 25; 30 min, A : B 50 : 50; 45 min, A : B 20 : 80; and 60 min, A : B 0 : 100. The mobile phase flow rate was 1.0 mL/min, the column temperature was 30°C, the injection volume was 10 *μ*L, and UV detection was at 230 and 330 nm.

### 2.4. Animals

Seven-week-old female Sprague-Dawley rats (180–200 g) were purchased from Orient Bio (Seongnam, Gyeonggi, Korea). Animals were maintained at a regular 12 h light/dark cycle, at 21 ± 2°C, with a relative humidity of 50 ± 5%, and were fed a commercial diet (Ralston-Purina, St. Louis, MO, USA). After 1 week of acclimation, rats were Sham-operated (saline; Sham-control group), or bilateral ovariectomies were performed under general anesthesia. The ovariectomized rats were randomly divided into 3 groups: (A) vehicle control (saline; OVX-control group) (B) aspirin 30 mg/kg/day (OVX-ASA group); (C) DJS 100 mg/kg/day (OVX-DJS group). The rats were orally administered the drugs for 5 weeks. Food intake was recorded daily, and the body weight was recorded weekly. The animals used in this study were treated in accordance with the Guide for Care and Use of Laboratory Animals by the Institutional Animal Care and Use Committee (IACUC) of Institute of Oriental Medicine (approval number: 14-003, 14-021).

### 2.5. Serum Lipid Levels

At the end of the experiment, rats were fasted for 12 h, anesthetized with Zoletil (1 mL/kg intraperitoneally), and sacrificed. Blood samples were collected from the inferior vena cava and centrifuged at 3000 rpm for 10 min to collect the serum. Measurements of TC, TG, and HDL-C levels were performed enzymatically using a Roche Modular P Autoanalyzer (Roche Diagnostics, Indianapolis, IN). The concentration of LDL-C was calculated using the Friedewald equation [12].

### 2.6. Measurement of Platelet Aggregation

Platelet-rich plasma (PRP) was separated from the blood using an anticoagulant citrate dextrose (ACD) solution that contained 0.8% citric acid, 2.2% sodium citrate, and 2% dextrose at a concentration of 3 × 10^8^ cells/mL. Platelet aggregation in response to collagen (2.5 *μ*g/mL) was performed in PRP using an aggregometer (Chrono-Log Co., Harbortown, CA, USA). The extent of aggregation was expressed as a percentage of the Sham-control stimulated with collagen.

### 2.7. FeCl_3_-Induced Carotid Artery Thrombosis Models

Saline or test compounds were administered 4 h before the initiation of thrombotic occlusion. During operative procedures, body temperature was maintained at 37.0 ± 0.2°C using a heating pad. After isoflurane anesthesia, one of the carotid arteries was exposed and a Doppler flow probe (Powerlab/8SP, ADInstruments Pty Ltd, Castle Hill, NSW, Australia) was placed on it. After stabilization for 3 min, vascular injuries were induced by a topical application of a FeCl_3_-saturated filter paper (2 × 2 mm). The filter paper was removed and the common carotid artery was washed with saline. A Doppler flow probe was placed on the exposed artery and blood flow was measured continuously with Laser Doppler Flowmetry (LDF; BFL21, Transonic Instrument, USA) for 30 min. To determine the time to occlusion (TTO), arterial occlusion was determined by decreased blood flow, and complete occlusion was defined as cessation of the carotid artery blood flow for 10 minutes.

### 2.8. Histology

The injured artery of rats was fixed in 4% paraformaldehyde and embedded in paraffin for histological analysis. These were sectioned longitudinally into 4 *μ*m slices and stained with hematoxylin-eosin (H&E) and Masson's trichrome. The thrombus for each animal was imaged using a light microscope (Axio Imager D2; Carl Zeiss Microimaging, Oberkochen, Germany) and the Zeiss AxioVision software (AxioVs40V4.8.1.0; Carl Zeiss imaging Solution) to obtain an image slice that represented the largest part of the thrombus.

### 2.9. Statistical Analysis

The results are presented as a mean ± standard deviation (SD). Statistical significance was analyzed using SPSS (version 20.0, SPSS Inc., Chicago, IL, USA). Differences among groups were compared with a one-way ANOVA test, followed by Tukey's post hoc test. *P* values less than 0.05 were considered significant.

## 3. Results

### 3.1. HPLC Analysis of Reference Compounds in DJS

**Figure 1:**
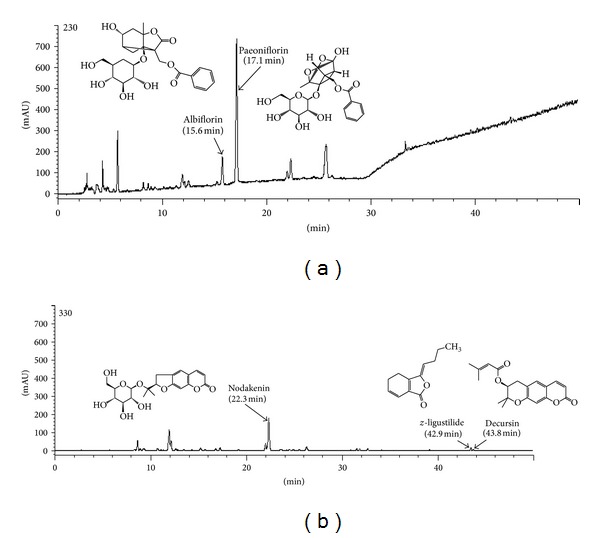
HPLC-DAD chromatogram of five reference compounds in DJS detected at 230 nm (albiflorin, paeoniflorin) and 330 nm (nodakenin, *z*-ligustilide, and decursin). DJS: Dangguijagyagsan aqueous extract (29.28 mg/mL).

The standard curves for the five components containing albiflorin, paeoniflorin, *z*-ligustilide, decursin, and nodakenin were *y* = 13.026*x* + 70.072 (*R* ^2^ 1.000), *y* = 13.446*x* + 124.71 (*R* ^2^ 0.999), *y* = 25.841*x* + 40.72 (*R* ^2^ 1.000), *y* = 38.995*x* + 31.219 (*R* ^2^ 0.999), and *y* = 19.043*x* + 108.95 (*R* ^2^ 1.000), respectively. HPLC analysis of DJS and standard mixtures was carried out at 230 and 330 nm. The retention time of each compound was 15.6 min (albiflorin), 17.1 min (paeoniflorin), 22.3 min (nodakenin), 42.9 min (*z*-ligustilide), and 43.8 min (decursin). The contents of each component in DJS aqueous extract were albiflorin 2.97 ± 0.065 mg/g and paeoniflorin 16.17 ± 0.120 mg/g at 230 nm, nodakenin 3.63 ± 0.005 mg/g, *z*-ligustilide 0.11 ± 0.004 mg/g, and decursin 0.020 ± 0.001 mg/g at 330 nm, respectively (Figure 1).

### 3.2. Effects of DJS on Body Weight Gain and Food Intake

The final body weights after 5-week treatments increased significantly in the OVX-control compared with that in the Sham-control group (*P* < 0.05). OVX-ASA and OVX-DJS groups showed similar body weight with OVX-control group. In the OVX-control group, treatment with saline demonstrated a significant increase in body weight gain compared to the Sham-control group (5.7 ± 2.4 g/day versus 3.7 ± 2.7 g/day, *P* < 0.001). The body weight gain was slightly decreased in the OVX-ASA (5.4 ± 2.4 g/day) and OVX-DJS groups (5.1 ± 2.2 g/day) compared with OVX-control group, although not significantly compared with the Sham-control group (*P* > 0.05). When animals were allowed free access to food following an experimental period, the food intake was the same in the Sham-control (17.02 ± 2.12 g/day) and OVX-control groups (17.72 ± 2.84 g/day). Food intake was increased in the OVX-ASA (19.14 ± 3.71 g/day) and OVX-DJS groups (19.39 ± 3.22 g/day) compared with the Sham-control (*P* < 0.001) and OVX-control groups (*P* < 0.01) (Table 2).

### 3.3. Effects of DJS on Serum Lipid Levels

**Figure 2:**
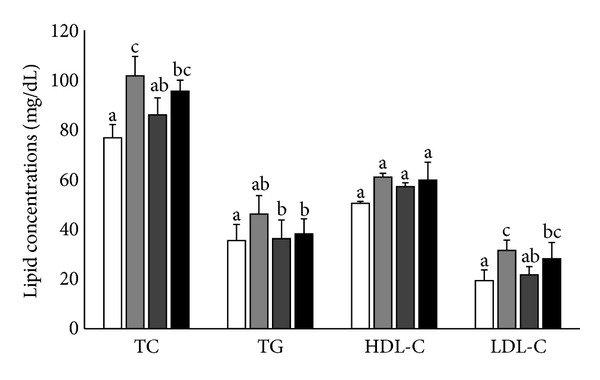
Effects of ASA (30 mg/kg/day) and DJS (100 mg/kg/day) on serum lipid profiles in SD rats compared with their corresponding controls: serum total cholesterol (TC), triglyceride (TG), high-density lipoprotein cholesterol (HDL-C), low-density lipoprotein cholesterol (LDL-C) levels in Sham (white bar) and OVX SD rats after 5 weeks of oral demonstration with saline (grey bar), ASA (dark grey bar, 30 mg/kg/day), or DJS (black bar, 100 mg/kg/day). Values of the same measured parameter that are not followed by the same alphabetical letter are significantly different (*n* = 5–7 in each group).

Ovariectomy caused a substantial increase in the TC, TG, and LDL-C levels compared with the Sham-control group (*P* < 0.001, *P* < 0.05, and *P* < 0.01). In OVX rats, treatment with 30 mg/kg/day of ASA (OVX-ASA group) and 100 mg/kg/day of DJS (OVX-DJS group) significantly attenuated the increase in the TC, TG and LDL-C levels but did not affect HDL-C levels compared with the OVX-control group (*P* < 0.05, resp.). Additionally, the OVX-ASA and OVX-DJS groups exhibited no significant changes in the HDL-C levels compared with the Sham-control and OVX-control groups (*P* > 0.05) (Figure 2).

### 3.4. Effects of DJS on Platelet Aggregation

**Figure 3:**
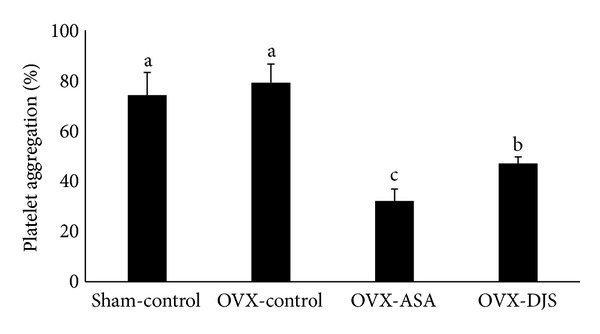
Inhibition by ASA (30 mg/kg/day) and DJS (100 mg/kg/day) of platelet aggregation induced by collagen (2.5 *μ*g/mL). Values of the same measured parameter that are not followed by the same alphabetical letter are significantly different (*n* = 5 in each group).

There was no difference in the inhibition of platelet aggregation between the OVX-control and Sham-control groups (79.5% ± 7.4% versus 74.5% ± 9.0%, *P* > 0.05). Treatment with 30 mg/kg/day of ASA markedly suppressed platelet aggregation (32.3% ± 4.7%, *P* < 0.001), as did treatment with 100 mg/kg/day of DJS (47.3% ± 2.6%, *P* < 0.001) compared with the OVX-control group (74.5% ± 9.0%) (Figure 3).

### 3.5. Effects of DJS in OVX Rats with FeCl_3_-Induced Thrombosis

**Figure 4:**
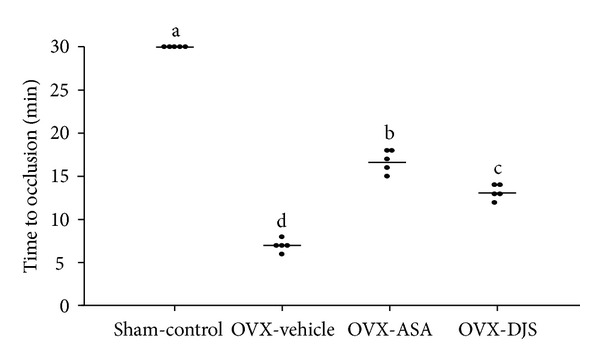
Time to occlusion of DJS in a carotid arterial thrombosis model. The horizontal lines represent the mean of the displayed values for time to occlusion in each group. Values of the same measured parameter that are not followed by the same alphabetical letter are significantly different (*n* = 5 in each group).

The mean time to occlusion in the OVX-control group was significantly decreased (7.80 ± 0.84 min) compared with that of the Sham-control group (30.00 ± 0.00 min). Therefore, the application of 40% FeCl_3_ to the external surface of the carotid artery for 3 min induced a rapid decrease in blood flow and was considered optimal for measuring blood flow. In comparison, treatment with either 30 mg/kg of ASA (OVX-ASA group) (16.80 ± 1.30 min) and 100 mg/kg of DJS (OVX-DJS group) (13.20 ± 0.84 min) also prolonged time to occlusion by 2.49 ± 0.33 ratio and 1.94 ± 0.08 ratio, respectively (Figure 4).

### 3.6. Histology of the Carotid Artery with Thrombotic Occlusion after FeCl_3_ Treatment

**Figure 5:**
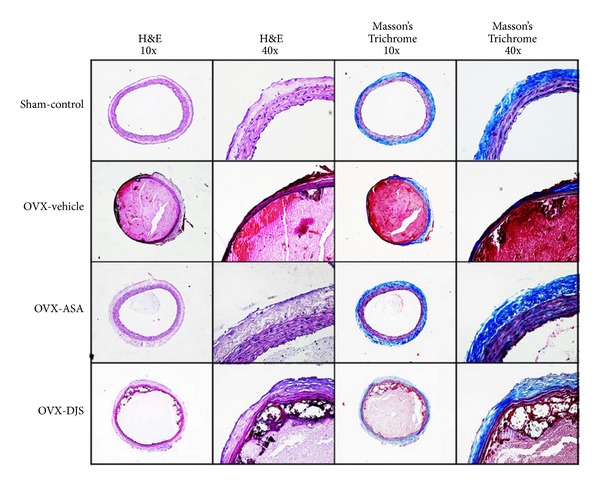
Histological examination of a FeCl_3_-induced thrombus from the Sham-control group, OVX-vehicle group, OVX-ASA group, and OVX-DJS group. All sections were stained with hematoxylin and eosin (H&E, two left columns, and magnification ×100, 400) and Masson's trichrome (MT, two right columns, and magnification ×100, 400).

Complete occlusion was defined as the absence of blood flow for 10 minutes after the application of FeCl_3_. We collected those arteries and processed them with H&E and Masson's Trichrome for collagen fiber staining. In this study, histological changes were observed in the carotid arteries after FeCl_3_-induced thrombosis. In these images, the type I collagen fiber stained blue, the nuclei stained black, and the background stained red. Thrombotic occlusion and collagen fiber damage were observed in the carotid artery of the OVX-control group after FeCl_3_ treatment. However, treatment with ASA and DJS provided an excellent recovery of collagen fiber in the thrombotic vessels induced by FeCl_3_ (Figure 5).

## 4. Discussion

Postmenopausal women frequently experience weight gain and are thus vulnerable to obesity-related diseases, which lead to an increased risk of cardiovascular diseases (CVD) such as coronary heart disease, hypertension, and non-insulin-dependent diabetes mellitus [13, 14]. In the present study, body weight gain was significantly increased in OVX-control group compared to Sham-control group for five-week treatment and there were no significant weight gain differences in OVX-ASA and OVX-DJS groups compared to OVX-control group. However, food intake in OVX-ASA and OVX-DJS group had much consumption compared to OVX-control group and these results suggest that administration of DJS could have beneficial effect on obesity in menopause.

Large number of pharmacological studies were carried out on the influence of the menopause on serum lipids and lipoproteins profiles. Menopausal statement increases the incidence of CVD due to hyperlipidemia, which is characterized by increases in total cholesterol (TC), triglycerides (TG), and low-density lipoprotein cholesterol (LDL-C) and a reduction in high-density lipoprotein cholesterol (HDL-C) [15–18]. Many attempts have been made to use herbal extracts to improve serum lipid levels in postmenopausal women suffering from hyperlipidemia associated with CVD [19–21]. In the present study, DJS treatment significantly reduced the TC, TG, and LDL-C levels (*P* < 0.05). Contrary to the conventional studies, HDL-C level of OVX-control did not appear to be significantly increased (*P* > 0.05). These results indicate that the rodent model for studying lipid metabolism physiology may have limitations, but it still provides a good model for studying the effects on serum lipid levels. Therefore, it is suggested that the effects of the DJS on serum lipid levels are very complex and should be investigated further. It has been reported that ovariectomy caused an elevation of TC and LDL-C in serum, leading to the development of atherosclerosis and coronary heart disease [22]. Furthermore, we further investigated the antithrombosis activity based on the serum lipid level changes of DJS treatment.

We also determined the effect of DJS on thrombus formation. Endothelial dysfunction is considered the first step in the atherosclerotic process, and our experimental approach was based on endothelial injury and platelet aggregation being key events in the pathogenesis of thrombus formation [23, 24]. Intravascular thrombosis is involved a series of complex events including platelet adhesion, activation, aggregation, granule release, and coagulation cascade activation, is likely to platelets contribute to thrombosis in several ways [25]. Platelets provide the membrane surface required for the generation of thrombin and express surface receptors that affect platelet-platelet and platelet-vessel wall interactions [26]. As a positive control, we used aspirin, one of the most widely used blood thinning agents, is used long-term in low doses to prevent heart attacks, strokes and blood clot formation in people at high risk for these events [27, 28]. In this study, although the cardiovascular protective effects of DJS treatment were not as strong as ASA, DJS treatment was successful in inhibiting platelet aggregation and thrombus formation. Previous studies have reported that biochanin A, a phytoestrogen that acts as a weak estrogen receptor agonist, restored endothelial function in an animal model of vascular injury [29]. This study showed the possibility that DJS treatment can restore endothelial function in an animal model of carotid artery injury, an effect similar to the vascular effects of estrogen.

Measurement of platelet aggregation has been a useful method to examine inhibition of thrombus formation. Furthermore, FeCl_3_-induced thrombosis model method in rats is the optimal technique as it exhibits simplicity and reproducibility. In a previous test, we demonstrated the arterial response to various concentrations (10–50%) of FeCl_3_ and found that filter paper saturated with a 40% concentration of FeCl_3 _resulted in fast, effective recanalization. FeCl_3_ triggers an oxidative vascular endothelial matrix. Then, platelets interact with collagen and vWF in the matrix via their respective platelet surface receptors, leading to platelet adhesion [30]. According to the* in vitro* results, the* in vivo* antithrombotic efficacy of DJS was anticipated. Indeed, the oral administration of DJS delayed the occlusion time in a FeCl_3_-induced artery thrombosis model. Notably, DJS doubled the occlusion time at 100 mg/kg compared with the OVX-control group. In this study, we did not design the Sham-vehicle and OVX-control groups to measure time to occlusion after the FeCl_3_ topical application. Our previous study investigated the effects of saline administration on rats following Sham operation and ovariectomy, which is similar to the Sham-control and OVX-control groups. In support of our hypothesis, the histological staining methods also demonstrated that DJS inhibited arterial thrombus formation and damage of collagen fiber in arterial vessel wall.

Various clinical studies have shown that herbal extraction components have significant association antioxidant effects; it leads to antithrombotic properties by increase of platelet NO release and radical scavenging activity [31, 32]. DJS consists of 6 herbs and each herb also has antioxidant effects [33–37]. We hypothesize that antioxidant effects are responsible for the results from above antithrombotic studies.

Female sex appears to be associated with cardioprotection and menopauses are major risk factors for cardiovascular disease. In fact, the ideal postmenopausal cardiovascular protective therapy is expected to reproduce the beneficial effects of blood flow without producing any adverse effects. Although the exact mechanism of action remains to be clarified, we have demonstrated the beneficial effects of DJS on the improvement of plasma lipid profiles and restoration of blood flow. These beneficial effects may be caused by (1) decreased TC, TG, and LDL-C; (2) inhibition of platelet aggregation; or (3) inhibition of thrombus formation. In conclusion, DJS could be a candidate for inhibiting the development of cardiovascular risk factors.

## Figures and Tables

**Table 1:** Composition of DJS aqueous extract and ingredients.

Ingredients	Contents (g)
*Paeonia lactiflora Pallas *	93.75
*Cnidium officinale Makino *	56.25
*Alisma orientale Juzepzuk *	56.25
*Angelica gigas Nakai *	28.13
*Poria cocos Wolf *	28.13
*Atractylodes japonica Koidzumi *	28.13

Total	290.00

**Table 2:** Effect of DJS administration on body weight gain and food intake in OVX rats.

Group	Final body weight (g)	Body weight gain (g/day)	Food intake (g/day)
Sham	306.83 ± 19.04^a^	3.74 ± 2.67^a^	17.02 ± 2.12^a^
OVX-control	341.15 ± 21.85^b^	5.72 ± 2.43^b^	17.72 ± 2.84^a^
OVX-ASA	345.91 ± 19.93^b^	5.39 ± 2.43^b^	19.14 ± 3.71^b^
OVX-DJS	346.54 ± 23.62^b^	5.11 ± 2.18^b^	19.39 ± 3.22^b^

Results are mean ± S.D. (*n* = 15). Means of letters recorded as a and b within a column indicated the same level of body weight and food intake within the values determined by one-way ANOVA. OVX: ovariectomized; ASA: aspirin; DJS: Dangguijagyagsan.
