# Corrigendum to “Effects of an Aqueous Extract of Dangguijagyagsan on Serum Lipid Levels and Blood Flow Improvement in Ovariectomized Rats”

**DOI:** 10.1155/2016/7249813

**Published:** 2016-10-31

**Authors:** In Sil Park, Hye Won Lee, Jin Ah Ryuk, Byoung Seob Ko

**Affiliations:** Korean Medicine Based Herbal Drug Development Group, Herbal Medicine Research Division, Korea Institute of Oriental Medicine, 1672, Yuseong-daero, Daejeon 305-811, Republic of Korea

 In the article titled “Effects of an Aqueous Extract of Dangguijagyagsan on Serum Lipid Levels and Blood Flow Improvement in Ovariectomized Rats” [1] there were errors, which should be corrected as follows.

In Table 2, the body weight gain and sample size are incorrect. The corrected table is presented here.

 In Figure 2, the sample size was not *n* = 5–7 but *n* = 4 in each group. The corrected sample size is presented here.

In the Abstract, “These effects were reduced by ASA and DJS treatment” should read “Other than body weight gain, these effects were reduced by ASA and DJS treatment.”

In the Results, “In the OVX-control group, treatment with saline demonstrated a significant increase in body weight gain compared to the Sham-control group (5.7 ± 2.4 g/day versus 3.7 ± 2.7 g/day, *P* < 0.001). The body weight gain was slightly decreased in the OVX-ASA (5.4 ± 2.4 g/day) and OVX-DJS groups (5.1 ± 2.2 g/day) compared with OVX-control group, although not significantly compared with the Sham-control group (*P* > 0.05)” should read “All OVX groups showed a similar body weight gain (OVX-control 3.82 ± 0.65 g/day; OVX-ASA 3.84 ± 0.60 g/day; OVX-DJS 3.79 ± 0.75 g/day) compared to the Sham-control group (2.94 ± 0.59 g/day).”

In the Discussion, “In the present study, body weight gain was significantly increased in OVX-control group compared to Sham-control group for five-week treatment and there were no significant weight gain differences in OVX-ASA and OVX-DJS groups compared to OVX-control group. However, food intake in OVX-ASA and OVX-DJS group had much consumption compared to OVX-control group and these results suggest that administration of DJS could have beneficial effect on obesity in menopause” should read “In the present study, body weight gain was significantly increased in all OVX groups compared to Sham-control group for five-week treatment. However, food intake in OVX-ASA and OVX-DJS group had increased consumption compared to OVX-control group.”

## Figures and Tables

**Figure 2:**
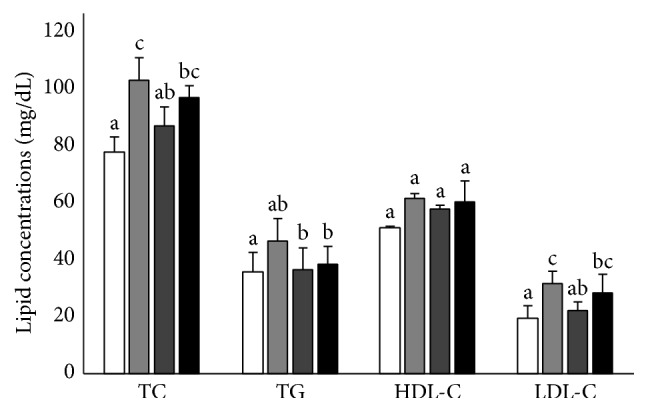
Effects of ASA (30 mg/kg/day) and DJS (100 mg/kg/day) on serum lipid profiles in SD rats compared with their corresponding controls: serum total cholesterol (TC), triglyceride (TG), high-density lipoprotein cholesterol (HDL-C), low-density lipoprotein cholesterol (LDL-C) levels in Sham (white bar) and OVX SD rats after 5 weeks of oral demonstration with saline (grey bar), ASA (dark grey bar, 30 mg/kg/day), or DJS (black bar, 100 mg/kg/day). Values of the same measured parameter that are not followed by the same alphabetical letter are significantly different (*n* = 4 in each group).

**Table 2:** Effect of DJS administration on body weight gain and food intake of OVX rats.

Group	Final body weight (g)	Body weight gain (g/day)	Food intake (g/day)
Sham	306.83 ± 19.04^a^	2.94 ± 0.59^a^	17.02 ± 2.12^a^
OVX-control	341.15 ± 21.85^b^	3.82 ± 0.65^b^	17.72 ± 2.84^a^
OVX-ASA	345.91 ± 19.93^b^	3.84 ± 0.60^b^	19.14 ± 3.71^b^
OVX-DJS	346.54 ± 23.62^b^	3.79 ± 0.75^b^	19.39 ± 3.22^b^

Results are mean ± SD (*n* = 10–12). Means of letters recorded as a and b within a column indicated the same level of body weight and food intake within the values determined by one-way ANOVA. OVX: ovariectomized; ASA: aspirin; DJS: Dangguijagyagsan.
